# Book Reviews

**Published:** 1817-10

**Authors:** 


### CRITICAL ANALYSIS

### *OF RECENT PUBLICATIONS,*

IN THE

### DIFFERENT BRANCHES OF PHYSIC, SURGERY, AND MEDICAL PHILOSOPHY.

---

*A Physiological System of Nosology, with a corrected and simplified Nomenclature.* By JOHN MASON GOOD, F.R.S.

*(Concluded from our last.)*

WE return with increased anxiety, and, if possible, increased diligence, to this important subject; and, as no former nosologist has ventured to write with decision on Elephantiasis, we have seen no reason to regret the accident which brought us to this genus.

"ELEPHANTIASIS.

"ELEPHANTIASIS.—Skin thick, livid, rugose, tuberculate; insensible to feeling; eyes fierce and staring; perspiration highly offensive.

1. ARABICA. Tubercles chiefly on the face and joints; fall of the hair, except from the scalp; voice hoarse and nasal; contagious and hereditary.

Elephas. *Dioscor.* lib. ii. *Lucr.* De Rer. Nat. vi. 1102.
Elephantiasis. *Aretæus.*
Elephantiasis. *Sag. Cull.*
Elephantiasis Indica. E. Orient. *Sauv.*
Elephantia Arabum. *Vog.*
Lepra Arabum. *Auct. Var.*
Juzam (——).
Khóra. *Hindust.* Asiat. Research. Vol. II. 8.
Elephanta-naussatz. *G.*
*Black Leprosy.*"

---

"Gen. IX. ELEPHANTIASIS. From ἐλέφας, 'an elephant:' so denominated by the Greek physicians, because the skin of persons affected with this disease resembles that of the elephant in thickness, ruggedness, insensibility, and dark hue. Thus applied, therefore, the term imports *elephant-skin,* in the same manner as the same national school denominated *dandriff* pityriasis *bran-skin,* from the skin under this disease resembling branny scales; and another sort of scaly malady ichthyiasis, or *fish-skin,* from the resemblance of the skin, when thus affected, to the scales of finny tribes.

"The Greeks became first acquainted with the elephantiasis from their casual intercourse with Egypt. To this quarter Lucretius, adopting the common opinion, ascribes its origin, lib. i. VI. 112:

Est ELEPHAS morbus, qui propter flumina Nili,

Gignitur Ægypto in mediâ, neque præterea usquam.

High up the Nile, 'mid Egypt's central plains,
Springs the BLACK LEPROSY, and there alone.

"Arabia, however, seems rather to have been the prolific source of this terrible scourge than Egypt, if we may judge from what seems highly probable, namely, that this is the *disease with which Job was afflicted* in Idumea, a part of Arabia, as described in the sacred poem that bears his name, and which affords, without question, the most ancient record in the world, composed in a mixt language of Arabic and Hebrew; and, if we add to this the still more powerful argument that the Arabic name of the disease has extended itself all over the east, and is almost the only name by which it is known in Egypt, Persia, and India, in all which regions the disorder is about equally common. Yet the Arabic name is not *elephas* or *elephantiasis,* but *juzam* (——), literally 'disjunction, amputation;' vulgarly, indeed, and more generally pronounced and written judam (——), and by the Turks judamlyk (——), from —— (judd), a root which imports 'erosion, truncation, excision;'

evidently referring to the destructive character of the disease, and the spontaneous separation of the smaller members, as the fingers and toes, when severe in its progress.

"In some parts of India, however, and particularly in Mysore, it is called also (and especially by the common people, who are frequent sufferers from it) *durda*, a Persian and Turkish term, applied to elephantiasis emphatically; for *durd* (——), in these languages, imports sickness, disease, or distress generally; whence *durda* (——) in Arabic, as well as in the two former tongues, is a common exclamation of distress—'væ!' 'prô dolor!'

"The Arabians, however, have a malady of a very different kind, to which they give the name of *elephas, elephantiasis*, or *elephant-affection*, in their own language dal fil (——), which is literally *morbus elephas;* and which they sometimes contract to fil (——), or elephas alone; so denominating the disease from its supposed resemblance to the dark, thick, and heavy leg of the elephant: as though *elephant-leg* instead of *elephant-skin*. It is the 'swelled, tumid, or Barbadoes leg' of modern writers. And, on this account, when learning, and especially medical learning, found an asylum, during the dark ages, at the splendid courts of Bagdat, Bassora, and Cordova, and the best Greek writers were translated into Arabic, or the best Greek and Arabic into Latin, two different diseases were found to possess a like name; for the Greeks, notwithstanding that they had already elephantiasis to signify *juzam*, could only translate *dal fil* by elephantiasis also. And hence arose a considerable degree of confusion, which has, indeed, continued to the present moment; for *elephantiasis* (——), dal-fil, or 'elephant disease,' is still applied by many writers to both these maladies; while not a few regard the two as nothing more than varieties of a common species, or species of a common genus. Yet the one is a *tuberculate affection of the whole body;* while the other is a scaly affection of only particular parts, and, commonly, of not more than a single limb. As a mere variety of the proper elephantiasis, the Barbadoes or elephant-leg, *buenemia* in the present system, occurs in the nosology of Dr. Young; while Dr. Parr, in his article ELEPHANTIASIS, confounds the two affections under one general character and history. In his article NOSOLOGY, indeed, he is not guilty of the same perplexity; for here he confines himself, under the term elephantiasis, to black leprosy alone, but at the expense of totally omitting the Barbadoes-leg or *dal fil*, which occurs no where in the range of his classification.

"The leprosy *(lepra Græcorum) the leuce* (λευκη) of the Greeks, and the *baras* or *beras* (——) of the Arabians, was, by many of the Arabian physicians, and very generally among the people, supposed in various cases to terminate in juzam or elephantiasis, as though these also were nothing more than different stages or degrees of the same disorder. And hence another error and perplexity in medical study. Alsahavarius thus unites them, and they are *jumbled together, or explained alike, in nearly all the oriental dictionaries;* in which beras or leprosy, and juzam or or elephant-skin, are, almost without an exception, regarded as convertible terms; beras being sometimes explained by the name of *mered* or *bald* juzam (the term mered, ——, signifying *bald*, i. e. pilis carens), and elephant-skin by that of *black beras*, a name occasionally applied to it by Avicenna. This oriental confusion of two different diseases was readily copied by the first Latin translators, till at length both in the east and west, beras or lepriasis, though literally *scale-skin*, became a sort of family name for almost every foul disfigurement of the skin, whether tuberculate or scaly, cutaneous or constitutional. And hence the *lazarettos* or hospitals established for elephantiasis or black leprosy during the dark ages, the *morbus Sancti Lazari* as it was also called, were filled with patients exhibiting blotches and blains of every kind; and are still denominated ——, (judam khaneh), or judum lazarettos.

"In the Linnéan system, elephantiasis and leprosy, and perhaps several other diseases, are included under the term *lepra:* all which the disciples of the Linnéan school, consistently with a principle already adverted to, ascribe to animalcules drunk in with the common beverage of water—and especially the Gordius *marinus*. The black leprosy is well described as it occurs on the borders of the gulf of Bothnia and in Finland, in an article entitled *Lepra* in the Amœnitates Academicæ, vol. iv. No. 31, by M. Uddman; in the course of which this opinion is plausibly and ingeniously supported. It is also supported by M. Tonning in art. 149 of the same volume, entitled *Rariora Norwegiæ;* and was, in fact, many years antecedently to this, suggested by M. Martin, also a pupil, and a very indefatigable one, of Linnéus, during a tour to Norway in 1758.

"At Madeira, and in some other parts of the world, elephantiasis does not appear to be contagious, nor perhaps hereditary. Yet the testimony of its being both, in the true elephantiasis or juzam of Arabia and the east, is so strong and concurrent among the Hindu Cabirajas, as well as the Arabic writers, that there can be no doubt upon the subject. The distinction is sufficient to make a variety of the former, if *future observation* should fully confirm the general opinion.

"Many of the characters here noticed are accurately and ably pointed out by Dr. Bateman in his 'Practical Synopsis of Cutaneous Diseases;' to which the reader may turn with great advantage for a fuller illustration of the subject."

If the reader, as directed, refers to Dr. Bateman's Synopsis, he will there meet with the following among the *notes* with which that work abounds.

"While this sheet was in the hands of the compositor, I was favoured by Mr. J. Mason Good, a gentleman distinguished by his knowledge of the oriental languages, with some observations relative to the original Arabic appellations of these diseases, which, while they confirm the views which I had entertained in general, *throw additional light on the subject.*

"'The

"'The Leprosy of the Arabs,' he says, 'appears to have been called by themselves immemorially, and is still called, *juzam* and *juzamlyk*, though vulgarly and more generally *judam* and *judamlyk*, from an Arabic root, which imports erosion, truncation, excision. The term *juzam* has passed from Arabia into India, and is the common name for the same disease among the Cabirajas or Hindu physicians, who also occasionally denominate it *Fisádi khún*, from its being supposed to infect the entire mass of blood, but more generally *khora*.'

"I learn also, from this communication, that the original Arabic term, which was used to denote the *tumid leg*, above mentioned, was *dal fil*, which is literally *elephant disease;* and, further, that '*dal fil* is the common name for the *swelled leg* in the present day among the Arabians, who sometimes contract it to *fil* alone, literally *elephas*.'

"But, although the Arabians in general distinguished the *juzam* from other diseases, yet I have observed that they sometimes mentioned the *baras (leuce)* as having an affinity with it, calling some forms of the juzam *black baras*. Mr. Good remarks, that '*juzam* itself has occasionally been employed in the same loose manner; and has been made to import leuce or vitiligo, as well as proper or black judam; though in the former case it is commonly distinguished by the epithet *merd*, i. e. *pilis carens*, as *merd-juzam*, *bald juzam*. The proper and more usual name for this last disease is *beras* or *aberas*, sometimes written *alberas*, though less correctly, as this last is *beras* with a mere prefix of the definite article.'

"Mr. Good adds, 'that one of the most celebrated remedies for this disease *(juzam)*, employed by the Cabirajas or Hindu physicians, is arsenic (*Shuce*, in India *sanc' hya)* mixed in pills with black pepper,' six parts of the latter being added to one of the former: the pills are ordered to be of the size of small pulse, and one of them is to be swallowed morning and evening, with some betel-leaf.

"Since the publication of the former editions, I have had an opportunity of seeing two cases of Elephantiasis, which have been under treatment in London during the greater part of the present year (1814); and in both the arsenic had been fully tried, and proved to be entirely void of any remedial power."—*Bateman on Cutaneous Diseases*, 3d edit. p. 310, 11.

As Dr. Bateman had previously collected more synonyms than we have leisure to count, some of which he retains, and others discards, the reader will determine what light is thrown on the subject by the addition of Mr. Good's. If it were admitted that the same disease has all these names, we shall not take upon us to decide, whether it is worth while for the student to learn, or rather to be puzzled with, them: And, if it differs in different parts of the world, and in different countries, we must either consider that several different ferent diseases are described without discrimination by the same numerous and undefined terms, or else that it really varies in different climates. Now, to suppose that the same disease has a different character at different periods of the world would be absurd, or would involve us in a question which never can be solved. If climate makes the change, it then becomes us to ascertain all the varieties, and the various climates to which each is assigned. The number of terms and of forms is sufficiently intricate in Mr. Good; but Dr. Bateman starts a positive contradiction, which he seems willing to reconcile by means which we have shewn must be unsatisfactory. Having settled that the disease is not infectious, his next difficulty is "With respect to the *libido inexplebilis;* the evidence of which is not so satisfactory. Its existence, however, (continues he,) is affirmed by most *modern* writers, with the exception of Dr. Adams. But Dr. A. observed, on the contrary, in the lazars of Madeira, an actual wasting of the generative organs in men, and a want of the usual evolution of them in those who have been attacked before the age of puberty. Is the elephantiasis of Madeira now less virulent than that at former times?—Has it undergone some change in its character?—Or, is the ancient account of the disease incorrect?" All this uncertainty might be excusable had elephantiasis been unknown in England, as in the days of Dr. Cullen. But, Dr. Bateman, we have seen, after all the difficulties which seem to beset him so thickly in the 1st and 2d editions, concludes the subject in the 3d by remarking, that, since his former edition, he has had an opportunity of seeing two cases of elephantiasis in London. After this, we might have expected an end of conjectures, and an accurate description of what was seen by the author.

The first person, we believe, who suggested a doubt concerning the contagious nature of Elephantiasis, was Dr. Heberden, of Madeira. Dr. Adams followed, and showed the limits which are fixed to its hereditary property. This was confirmed by Dr. (now Sir ——) Christie, in the East Indies; by Dr. Gourley, in Madeira; by Mr. Want, in the London Med. and Phys. Journal; by Mr. Laurence, in the Medico-Chirurg. Transactions; by Dr. Roberts, in the Medical Transactions of the College of Physicians in London; and, we believe, since Mr. Good wrote, by Dr. Lee, in the Medico-Chirurg. Journal. Yet one of the most remarkable symptoms of the disease, the wasting of the testicle, admitted by all these, accurately described by some, and even carefully noticed by three writers in a specimen seen by Dr.

Bateman,

Bateman, is introduced by that gentleman with some doubt,* and entirely passed over by Mr. Good.

In the last gentleman's preliminary Dissertation, we are informed that his object is "not so much to interfere with any existing system of nosology, as to fill up a niche still unoccupied in the great gallery of physiological study." A disease, therefore, unknown in England when Dr. Cullen wrote, and which, on that account, he scarcely ventured to describe, had a fair claim to this vacant space, and we can only regret that it should be occupied by words in the interpretation of which every dictionary will deceive or puzzle us, sometimes referring to a white, sometimes a black, sometimes a scaly, sometimes an elephant's or a tubercular skin, or to an elephant's leg. It may be a mark of industry, but it cannot be called of perspicuity, when we are directed from Norway to the Indian Ocean, taking Persia, Asia minor, Egypt, and Arabia, in our route, for the description of a disease which has occurred twice in St. Bartholomew's Hospital, and been described by half a dozen living witnesses.

But this niche is so overloaded, that Mr. Good must find some means of relieving it, if he has any mercy on the building, or on those who study it. The lepra Græcorum, we are told, was the *leuce*, λευκη, of the Greeks,—and this in the same note in which we are referred to Dr. Bateman for the "great advantage of a fuller illustration of the subject." Now Dr. Bateman, in about a *dozen* different *notes*, assures us that *leuce* was not lepra Græcorum, but the leprosy of the Jews. Thus, logically reasoning, we arrive at the account of leuce, and of the leprosy of the Jews, both which have so often eluded our research in Dr. Bateman: for, if *leuce* is the leprosy of the Greeks, and also the leprosy of the Jews, then the whole is resolved into lepra Græcorum, a disease occurring every day in London. We admit that a difficulty still remains from the contradiction of these two learned writers, but we are truly glad to compromise the matter as well as we can. All this, the reader will keep in mind, relates only to *leuce*. We have still much to understand concerning Elephantiasis. Of this both these writers are so sensible, that Mr. G., as we have seen, waits for *future observation;* and Dr. B. concludes his account by remarking, that "accurate histories of Elephantiasis and *leuce*

* We have just learned that Dr. Bateman has published a fourth edition, without altering this passage. When discoveries are thus stifled, and errors continued in works which we wish to consider as class books, is it possible that medical science can be progressive?

are still among *the desiderata of* pathologists." Who can believe, after this, that leuce is only *lepra Græcorum;* or that Dr. Bateman has actually seen with his own eyes two cases of Elephantiasis?

The manner in which the notes are arranged brought *syphilôdes* before our eye, and, of course, directed our attention to syphilis, with which that *genus* commences: and here we conceived was another fair opportunity of filling a niche.

"*Lues.*—Ulcers on the genitals, inguinal buboes, or both, after impure coition; succeeded by ulcers in the throat, copper-coloured spots on the skin, bone-pains, and nodes.

Syphilis. *Sauv. Young.*

*Venereal Disease.*

1. SYPHILIS. Ulcers on the genitals circular, ungranulating, thickening at the edge; those of the throat deep and ragged; symptoms uniform in their progress; yielding to a course of mercury; not known to yield spontaneously.

Syphilis. *Linn. Vog. Sag. Cull.*

Syphilis venerea. *Sauv.*

Syphilis maligna. *Young.*

Lues venerea. *Astruc. Macbride.*

Frenk zchemeti (——).

Frantzosen. *G.*

Vérole. *F.*

*Pox.*

The above Arabic is literally Morbus Gallicus: but the more general name is —— (nar farsi), or —— (ateshi Farsi), according as the Arabic or Persian language is made use of; both meaning equally Ignis Persicus, or Persian Fire. By this name it is constantly denominated by the Cabirajas or physicians of India.—The name is probably taken from Avicenna, who thus distinguished the malignant carbuncle to which syphilitic eruptions are *supposed, in the east,* to have a near resemblance. See Phyma Anthrax.

2. SYPHILÔDES. Ulcers indeterminate in their character; symptoms irregular in their appearance; usually yielding spontaneously; variously affected by a course of mercury.

*Pseudo-syphilitic Disease. Abernth.*

Syphilis pseudo-syphilis. *Young.*

*Sibbens. Sivvens.* Qu.?

The varieties seem to be numerous; but have not, hitherto, been sufficiently defined for classification."

---

"Gen. VIII. LUES. From λύω, 'solvo, dissolvo,' 'to macerate, dissolve, or corrupt.' Agreeably to the common rule of expressing the power of the Greek υ by a Roman *y*, this should be written *lyes;* but the contrary has obtained so long and so generally, that it would be little less than affectation to attempt a change.

"That acrimonious and poisonous materials are at times secreted by the genitals, capable of exciting local, and, perhaps, constitutional affections in those who expose themselves to such poisons by incontinent sexual intercourse, appears to have been known to the world from a very early age. Celsus enumerates various diseases of the genitals, most of which are only referable to this source of impure contact: but the hideous and alarming malady which was first noticed as proceeding from the same source towards the close of the fifteenth century, and which has since been called almost exclusively *venereal disease*, has suppressed, till of late, all attention to these minor evils, in the fearful contemplation of so new and monstrous a pestilence; to various modifications of which most of the anterior and slighter diseases of the same organs seem to have been loosely and generally referred; as though there were but one specific poison issuing from this fountain, and consequently but one specific malady.

"The keen and comprehensive mind of Mr. John Hunter first called the attention of practitioners to the idea of different poisons and different maladies; and the subject has since been pursued by Mr. Abernethy with a force of argument, and illustrated by a range of examples, that seem to have put the question at rest. The latter has sufficiently established, that, independently of the specific disease now generally recognized by the name of syphilis, there are numerous varieties of some other disease, perhaps other specific diseases, which originate from a distinct, possibly from several distinct, poisons, secreted in the same region from peculiarity of constitution, or causes hitherto undiscovered; and which are accompanied by primary and secondary symptoms that often vary in their mode of origin, succession, and termination, from those of genuine syphilis; though, in many instances, they make a striking approach to it: and to which, therefore, Mr. Abernethy has given the name of *pseudo-syphilitic diseases*.

"Whether these really constitute distinct species of disease, issuing from distinct sorts of infection, or are mere varieties or modifications of one common species produced by one common morbid secretion, has not yet been sufficiently determined. In this ignorance upon the subject it is better, for the present, to regard them in the latter, as being the more simple, view;—the author has hence given to this species the name of *syphilodes*, and has arranged it, along with *syphilis*, under the generic term *lues*. The sibbens or sivvens of Scotland seems to appertain to this species.

"The origin of the term *syphilis* is not exactly ascertained. Dr. Turton spells it *siphilis*, and derives it from σιφλος *(siphlus)* 'foul or filthy;' Sauvages, from σὺν *(syn)*, 'together,' and φιλέω *(phileo)* 'to love,'—'*mutual love*:' in which case it should be spelt according to the common rule. The latter, who introduced the term into the nosological catalogue, appears to have derived it from Fracastoro, who was born at Padua in 1483, and died in 1553, and who spells it, in his very elegant poem *De Syphilitide*, upon this very inelegant subject, agreeably to the usual mode. There is an equal uncertainty certainty as to the quarter in which the disease originated. It is usually ascribed to the American continent, and believed to have been imported into Europe by the crews of Columbus, on his return home: a belief, however, which seems to be altogether without foundation. The reader who is desirous of examining the authority on which this opinion rests, together with the various names by which it was at first recognized, may consult the author's article on Medical Technology, in the Transactions of the Medical Society of London, series ii. vol. i. p. 21 and following, and the notes there subjoined.

"Linnéus stands alone in arranging syphilis as an exanthem, along with small-pox and measles. He thought himself justified from the fever which occasionally accompanies the copper-coloured spots on the skin, in an advanced stage of its secondary symptoms.

"In the Amœnitates Academicæ, vol. iv. art. 72, entitled 'Specifica Canadensium,' by M. Von Coelln, we have an exact formula for exhibiting the lobelia *syphilitica*, or Indian specific for syphilis, as delivered to Sir William Johnson, who purchased it of the Indians at a high price.

"Among the Hindus, the disease is commonly known by the name of *Persian fire;* a term, however, of much earlier date than any records we possess of the existence of syphilis, and applied by Avicenna, and the writers of the Saracenic schools, to a particular species of anthrax. The Hindu specific for the cure both of syphilis and elephas is a composition of arsenic, *sanc' hya*, in Arabic *shucc*, but which the Persians call *mergi-músh*, or mouse-bane. The oxyd employed is the white arsenic, a. *calciforme*, and the following is the mode in which the medicine is prepared, as given in the Asiatic Researches, vol. ii. art. viii. 'Take of sanc' hya *(white arsenic)* fine and fresh one tólá, (equal to 105 grains); of picked black pepper six times as much; let both be well beaten at intervals for four days successively in an iron mortar, and then reduced to an impalpable powder in one of stone with a stone pestle, and thus completely levigated, a little water being mixed with them. Make pills of them as large as tares or small pulse, and keep them dry in a shady place. One of the pills must be swallowed morning and evening with some betel-leaf; or where betel is not at hand, with cold water. If the body be cleansed from foulness and obstruction by gentle cathartics and bleeding, before the medicine is administered, the remedy will be the speedier.' The Cabirája, or Hindu physician, relies implicitly on the virtues of this composition, and confidently predicts the most certain success in both the above diseases. The cathartic previously employed is commonly manna, which is worked off with copious draughts of a cooling decoction of the *nilúfer* or nymphæa nelumbo.

"See also Sir William Jones's Works, vol. i. p. 553, 4to. edit."

It is certain that the keen comprehensive mind of Mr. Hunter first called the attention of practitioners to the number of morbid poisons which affect the genitals, the lips, and

 other other parts covered with a thin cuticle, and exposed to contact with the secretions of another person. But that same comprehensive mind fixed the exact character of the venereal ulcer, and also of several others. Without assigning any name to the latter, he marked those peculiarities in which they differ from the former, and from each other. As we are no where told what forms a genus, what a species, and what a variety, we may be allowed to express a wish that Mr. Good had satisfied himself with referring us to Celsus and Hunter. The former, in his "Chapter de Obscænarum partium vitiis," describes, with the greatest accuracy, every disease on those parts excepting the venereal ulcer; and Mr. Hunter, after a most minute description of that ulcer, concludes with an account of "diseases resembling the venereal, which have been mistaken for it." In this he almost translates the above chapter of Celsus, though it is well known that he was unable to read him; and such must ever be the coincidence between writers who describe from nature only. To what purpose, then, is the accuracy of these writers obscured by the introduction of words ill understood, or by referring to diseases on which we can scarcely form a conjecture. To what purpose are we told of the virtues of the *lobelia syphilitica*, when we are ignorant of the disease for which it is found a remedy; or why are we still more confused with the Persian fire,—a term older than syphilis? or to what purpose have we a receipt for syphilis and elephas, when we know that the remedy prescribed will cure neither the venereal disease nor elephantiasis. This is the part of the work which we view with the most regret. Mr. Hunter's comprehensive mind is, indeed, admitted, but none of his nicer discriminations are so much as hinted at. Even his term—"morbid poisons,"—now adopted by Jenner, Abernethy, and all who wish to be particularly correct, is passed over; and the whole concludes somewhat like the last mentioned genus, with a remark that "*the varieties have not been sufficiently defined for classification.*" It is very probable they never can be for *artificial arrangement*, as Dr. Bateman has also lamented. But it would be dreary indeed if we are to remain nothing better for the labours of Celsus and Hunter. We shall therefore conclude with transcribing an arrangement, not a classification, attempted agreeably to the language of the one, and the discriminating observation of the other.

The following is the conclusion of the chapter on the local Characters of Morbid Poisons, in a work alluded to in our Collectanea.

"If

"If this distinction of the local actions induced by morbid poisons be admitted, it will resolve itself into the following division:

Encreased and altered secretion on secreting surfaces, without loss of substance.

——————————————— non-secreting surfaces, with loss of substance, viz.

1. Slough, with consequent fungus and scab, as in yaws.
2. ———, with suppuration and scab, as in small-pox.
3. ———, preceded by ulcer, and when separated followed by immediate skinning, as in several anomalous poisons.
4. ———, with ulceration, and each in succession, as in the sloughing phagedæna.
5. Ulceration, kept up by the irritation of the secreted pus, as in sivvens and some anomalous phagedænæ.
6. ————, with a thickened edge and base, as in the venereal."

Thus it appears, that, if these eruptions, to use Dr. Bateman's expression, bid defiance to [artificial] arrangement, they admit of descriptions according to nature. Happy the students who, from this time, are relieved, in this instance at least, from nosological enigmas, and directed to the study of nature. This is still more remarkably illustrated in the succeeding extract.

We could not dismiss a nosological article without turning to typhus. Here again the index deceived us, and brought us to

"Order II. Phlogótica.—*Inflammations.*—Fixed heat and pain, or soreness; increased secretion, lesion of a particular part or organ; mostly accompanied with fever."

---

"Order II. Phlogótica. From φλέγω, 'incendo, ango.' Linnéus, for this order, employs *phlogistici*, from the same root; but, as the chemists have long since laid hold of *phlogiston*, and the term, though lately disused, has a chance of being restored, the author has preferred the derivative now offered. Cullen has *phlegmasiæ*, after Galen and Sauvages: but, as *phlegmatia* and *phlegmatic*, from the same source, import, in common medical language, very different, and almost opposite, ideas, this term is also purposely relinquished, to prevent confusion.

"Sauvages divides inflammations or phlogotic diseases (with him phlegmasiæ) into exanthematous, membranous, and parenchymatous: Linnéus into membranous, visceral, and muscular; referring the exanthematous diseases to another class. Cullen has disapproved of both these modes of division, as conceiving it difficult to ascertain the seat of the affection.

"The whole of the observations of Mr. John Hunter upon this subject are worthy of being deeply studied; and will not a little elucidate the nature of the arrangement introduced into the present method. It may be sufficient to observe, that, in treating on inflammation, flammation, he divides the body into two parts: 1, the circumscribed cavities, organs, and cellular membrane which connects them; and 2, the outlets of the body, commonly called mucous membranes, as the ducts of the glands, alimentary canal, &c. p. 240, 241, 254. He distributes inflammatory affections into three kinds, adhesive, suppurative, and ulcerative. Adhesive inflammation belongs chiefly to the former of the above two parts of the body, *where they are deeply seated;* and appears intended to take place in order to prevent suppuration. It applies, therefore, peculiarly to the genus *empresma,* in the present order, except in gastritis, enteritis, and cystitis; in all which, however, we frequently meet with striking examples of the adhesive inflammation, or true empresma, insomuch that the affected organ becomes at times so closely united with some adjoining membrane or other organ, as to obtain a kind of artificial wall or paries, and prevent the escape of its contents into another cavity, when ulcerated through the whole thickness of its substance. See the note on empressa, species 10. α. Suppurative inflammation belongs chiefly to the same division of parts, *placed near the surface—Hunter,* p. 252; and consequently applies peculiarly to the two genera of phlegmone and phyma. The ulcerative belongs chiefly to the second order of parts, p. 254, 255, as the mucous membranes and outlets; and hence principally applies to the genus erythema, as it must also be allowed to do to that of phlysis. Deep-seated suppurative inflammations and abscesses cannot well be placed in either of these genera, and have a claim to be considered by themselves: they are hence included in the genus apostema, with which the order opens.

"Dr. Young, if the author understands him rightly, unites the phlegmone and empresma into one and the same *species* of inflammation, inflammatio phlegmonica, of which he makes them only different varieties. Phlegmone was, indeed, used with this latitude among the Greeks, for it imported inflammation generally; but it has long been limited to suppurative, and by most writers to subcutaneous as well as suppurative inflammations, or those immediately under the skin, and those too in which the suppuration is perfect, and occupies the whole cavity. It is possible, however, that the author may not have understood Dr. Young aright, as he afterwards makes the different species of what is here called empresma, species of inflammatory fever or cauma.

"Phlegmon then, in the present method, is used to denote an inflamed subcutaneous tumour, perfectly suppurative; phyma, an inflamed subcutaneous tumour, imperfectly suppurative; ionthus, a subcutaneous tumour or tubercle, slightly inflamed, hard, and insuppurative; and phlysis, an inflamed, but low and broad tumour, ulcerative, exquisitely painful, and running among the tendons, of which the paronychia or whitlow is, perhaps, the only known species. The paronychia, as Galen has justly observed, has an approach to the erythema, or erysipelatous inflammation, which immediately follows it, but the two must not be intermixed."

We have often heard Mr. Hunter accused of obscurity, but, on this this occasion, we see him referred to by Mr. Good to "elucidate the arrangement introduced into the present method." We are much pleased with this remark, if it means, as we presume it must, that, after studying artificial arrangements, and nosological methods, we shall meet with the clearest information by carefully attending to Nature and Mr. Hunter.

We had prepared remarks on several other *genera*, but it is time to conclude.

Having often expressed our opinion of Nosology in general terms, we gladly seized this opportunity of discussing the claims of an experienced, as well as a learned, practitioner. Most of the later systems have been the production of younger writers, of whom it may be delicate to say, that they were scarcely aware of the magnitude of their undertaking. It will, perhaps, be asked, Is medicine the only science which cannot be reduced to system? To this we answer, that system, in all the productions of nature, is artificial, and consequently, in some points, erroneous, but attended with little danger in any but medicine. The first inquiry is, What is intended by a system? The answer is not difficult:—Without certain marks which we perceive in what we chuse to denominate a class, a genus, and a species, the whole of natural science would appear without order: it would be difficult for philosophers to understand each other, or for learners to direct their inquiries. This must be admitted; but every philosopher has also admitted the difficulty of ascertaining the characters by which his genera are to be distinguished, and the imperfections of all: they have, therefore, contented themselves with the best, in doing which, they have selected certain permanent marks, which may be viewed, and must be admitted by every diligent inquirer into nature. Is such the case in the arrangement of diseases? To a certain degree, it may be answered. Then let us extend our systems to that degree, and no further. That certain diseases have been accurately characterised by the genius, diligence, and faithfulness of different authors, has already been admitted, and, besides those before enumerated (see p. 229 of our last Number), we might add Heberden for angina pectoris, Hey for fungus hæmatodes,* Jenner

* The manner in which Mr. Good has disposed his notes, or "running commentary," as he very justly calls them, has frequently induced us to read backward. Thus, the notes on Elephantiasis, commencing under the genus Syphilis, led us to Lues; and, by the same incident, the notes on lues have led us to Cancer. On this our

Jenner for the vaccine vesicle. To Dr. Hume we are indebted for the first discrimination of croup, as it appears in infants. The same disease, in more advanced life, has now been ascertained, with no other difference than such as we might expect from the difference of age. For the discovery of the disease in infants we are not indebted to nosological tables, but to the observation of the unlearned, insomuch that the vernacular names are still retained in Britain and in America. The disease in advanced life, though accurately described by the ancient Greek and Roman writers, and spoken of by Sydenham as by no means uncommon, seemed almost unknown in our own nosological age when we had to lament the loss of two most respectable physicians. Was it by nosological tables that Dr. Heberden, even without the assistance of morbid anatomy, first taught us to distinguish angina pectoris from other forms of asthma; and afterwards, with a modesty characteristic of the man, placed it alphabetically as pectoris *dolor*, thus making the *pain*, as it is the most prominent, so the leading, character of his disease.

When we first viewed Mr. Good's performance, we expected, indeed, to meet with every disease in the organs, in the order he has arranged them. But we flattered our hopes also with a renewal of the important division of diseases into chronic and acute, and in the subdivision of each organic disease, with a detail of those symptoms which require the most immediate attention. This induced a wish that he had commenced with inflammation, because the general treatment would be similar in all such cases, though the sympathies, as well as symptoms, often vary. When inflammation is accurately defined, its progress traced, and its consequences pointed out, the first and most urgent part of medicine is accomplished. This is a grand desideratum in fevers, and it is truly astonishing that no nosologist before Dr. Young should have seen the necessity of placing inflammation anterior to fevers. In Boerhaave's days, morbid anatomy had not been sufficiently cultivated to teach us how much was to be apprehended from inflammation in fever of every description, and of every name. The same may

---

our remarks were very copious; and, as we could not compress them, we have omitted the whole, excepting one piece of justice which we cannot withhold from Mr. Hey. With what astonishment will the reader learn that only a single name is referred to in the division β spongiosus, or fungus hæmatodes, and that the name is not Hey. We omit its insertion, because we are sure it must offend the modesty of the person named, and that Mr. Good must regret his own inattention.

be be said almost to the time of Cullen, before which we had only the solitary facts which Pringle and a few others afford us. But later information taught Dr. Young that, in fevers, inflammation is the first consideration. Not only in fevers, but in all local diseases, especially if attended with heat and pain. By an inattention to this, Mr. Good has overlooked the most formidable, and, probably, the most remediable, of all the complaints in his first genus of Odontia, though he had the example of Mr. Hunter for his guide. After a general view of the peculiarities of the teeth, and of the diseases to which they are liable from that peculiarity, the attention of his "discriminating mind" is directed to inflammation, its symptoms, and mode of treatment, from a conviction, that, when extended to any great degree, it is productive of mischief which we have afterwards no means of relieving.

The mention of pain in Dr. Heberden's Nomenclature, leads us back to Mr. Good's preliminary dissertation. In the early part, he tells us that the only method for a nosological system worthy of attention is built on distinctive *symptoms*, and afterwards symptoms, if not the disease, are considered as algebraical characters leading to a knowledge of the disease. Yet, in a subsequent part, "the *family* of DOLORES, or LOCAL PAINS, is," we are told, "entirely suppressed, and the genera or species of which it has ordinarily been composed are distributed, as *mere symptomatic* affections, under other heads." In this manner, it is added, Cullen found it necessary to conduct his system; and Dr. Parr, after asserting the necessity of an *order*, at least of DOLORES, abandons it when describing his method. And what is the consequence? That "Cullen makes Odontalgia a species of rheumatism," and Parr "omits all notice of Cephalalgia, or even of Hemicrania."

We shall not venture into the inquiry whether DOLOR is consistent with an artificial order, but no one will doubt its physical existence, or that, in a practical point of view, it is the first thing to be attended to. Even its absence is often an important consideration: but, when present, can a practitioner be excused if he does not direct his first attention to its degree, its probable seat, and, most of all, to its relief. It may be said that the part of which our patient complains is often merely sympathetic. Does not that very circumstance prove the high importance of studying pains? It is enough to remark, that, wherever we have pain, we have reason to apprehend inflammation, and such we shall see, if not the opinion, was the practical inference, of writers whom we affect to venerate. To a modern nosologist, or writer of systems, it may seem strange that Celsus should, in his second book, give a chapter concerning the treatment of diseases *(de morborum curationibus)*, and afterwards that his 3d book should commence *de morborum generibus*. But nothing, in our opinion, can be more judicious than first to make us aware of those *indicia* or symptoms which, he observes, are common to many diseases, and indicate immediate *agenda*. Among others, his rules for blood-letting are so well marked, that we are forced to regret how often they have been overlooked since the introduction of nosological systems. "Ergo vehemens febris, ubi rubet corpus, plenæque venæ tument, sanguinis detractionem requirit: item viscerum morbi, nervorumque resolutio, et rigor, et distentio: quicquid denique fauces difficultate spiritus strangulat; quicquid subito supprimit vocem; *quisquis intolerabilis dolor est;* et quacumque de causa ruptum aliquid intus atque collisum est."

In these comprehensive lines we have, in our opinion, instructions more important to the young practitioner, and more serviceable to mankind, than are to be met with in volumes of nosology. The symptoms of fever in any stage—visceral diseases, whether attended with pain or not—apoplexy—the severe forms of quincy, be the seat of the disease in whatever part of the throat—and violent pain, from whatever cause,—demand the immediate use of the lancet. Such aphorisms are, indeed, practical; but we have yet to learn what are the *practical* advantages of nosology.

Such, then, is our opinion of nosology, and such appears, sometimes, Mr. Good's opinion of all nosologies excepting his own. He must not, therefore, take it amiss if "future nosologists (should the present work have any pretensions to futurity)" amuse themselves as he has done. We have already, without design, extracted several passages in which Mr. Good is very pleasant on his predecessors. Another obtruded itself upon us, whilst, under the article Inflammation, we were searching for a methodical arrangement of Tracheitis or Laryngitis. It shows how easy a thing it is to make nosology ridiculous, so that we cannot help advising any future undertaker of this modern art to reflect on Horace's caution—

> Quid rides? mutato nomine, de te
> Fabula narratur.

"Dr. Cullen (we are told) is said to have prided himself upon having grouped an extensive natural family of diseases under the term cynanche. Parr, art. ANGINA *stridula,* denies that he has done so; and adds, that 'self-complacency had never so baseless a foundation. The species,' continues Parr, 'agree in no one principle ple but affection of the parts connected with the neck.' This remark is too sweeping: it may apply to Cullen's species of c. trachealis (croup), and c. parotidœa (mumps); but the rest must be exempted from its severity. And it is not a little ludicrous to observe Parr, after passing this censure in his article *Angina,* under which he considers the disease in its different bearings, completely altering his views by the time he reaches the article *Nosology:* for here we find, in the first place, the term *angina* banished, and that of *cynanche* adopted in its stead; and secondly, croup and mumps, which are chiefly objected to in the preceding quotation as divisions of *cynanche,* not only made divisions, but sunk from species, in which they occur in Cullen, into varieties. The present arrangement follows the Cullenian as far as, perhaps, it ought to do, and only quits it where the latter seems to demand a change."

All this is very entertaining; but we must leave those to enjoy it who consider physic a joke, and who can so soon forget the names of Pitcairn and Macnamara Hayes. That Mr. Good is an able practitioner and a profound scholar, we cannot question. His good sense, his age, and the department he has chosen, must have entitled him to the first character; and his quotations from all languages satisfy us concerning the second. But either we are much mistaken, or he has attempted what cannot be accomplished in the present state of medicine, and, though we have given him credit for the diligence he has shown in correcting our nomenclature, yet we conceive that his attempts at simplicity have sometimes deceived him. When we expressed our satisfaction at not meeting with pseudos or oïdeses among the affixes or suffixes, we little expected to meet with syphilòdes used in a sense similar to the syphilòides of other writers. Whether these suffixes are both from the same root, it is not our province to inquire; but that they are used in meanings not precisely the same, will not, we suspect, be questioned. *Oïdes* is well known to be formed from εἶδος, *forma,* and is often convenient in anatomy and other branches of natural philosophy when used with great caution: *odes,* on the contrary, when joined to a Greek substantive, makes an adjective, which may be used in the more general sense expressed by "of or belonging to." In this manner Mr. Hey has named the disease so accurately described by him, and, with equal propriety, often called "bloody cancer." But, had he called it *hæmatoïdes,* it would be likening a thing to itself. We know not in what way to treat Mr. Good's syphilodes. An ulcer, "indeterminate in its character, irregular in its symptoms and appearances, usually yielding spontaneously, and variously affected by mercury," can never *be like,* or have *any kin* to,

 an an ulcer of a determinate character, "yielding to mercury and not known to yield spontaneously." The only kindred is locality, which is scarcely less "consistent with professional gravity" than psora and broken bones, or the other locales of Dr. Cullen. (See p. 213 and 214 of our last Number.)

Many other instances have occurred in which Mr. Good appears to us to have sacrificed accuracy to simplicity. Let us refer to the specimen transcribed from the preliminary dissertation into the beginning of this article (see page 218, which we request the reader to re-peruse). Here we are told that *mariscæ*,—in Latin, hæmorroides, and *γαζα* in Greek, are the same thing. That the root is Arabic (——Khazan) or Persian, or both, to us it signifies little which. But we shall take the liberty, if not to set Sauvage and Mr. Good right, at least to differ from them.

These *mariscæ* have been well known from the time of Astruc by the name of *fici*, and very probably each name is derived from a similar property. Martial, Mr. Good informs us, speaks of them as figs: the Greek term is, we believe, *συκωμα* or *συκωσις*. Homer, in his account of the garden of Alcinous, tells us that figs grew in such succession that some were constantly ripe—*συκον επι συκω γηρασκει*. This constant succession, with a spongy, succulent, and sticky property, it is supposed gave rise to the term *συκοφαντης*, sycophant; at least it helped Aristotle to his pun on quitting Athens, whilst the citizens were rejoicing at the death of Socrates. Thus, gentle reader, you perceive that we, now and then, venture to stray as far as Aristotle; but we pretend not to know whether his *gazæ* were *mariscæ*, or whether either or both were *hæmorrhoides*: nor do we venture to dispute with Hesychius, or Scaliger, or Mr. Good, whether *γαζα* is a Persian word, and means treasure, or whether it is rather an Arabic than a Persian term—whether it signifies redness, blushing, fulness, or autumn, &c. &c. Yet we may be allowed to remark, that, though *podix* and *anus* are sometimes used in a very general sense, each had its appropriate meaning. The poets, when speaking to the first of these terms, never refer to the latter. These mariscæ were on the podix, not growing from the extremity of the rectum. We shall not refer to the Latin derivation of mariscæ given by some commentators on the poets, any further than to shew that in those days an idea of the inoculation of morbid poisons seems to have prevailed (see Juvenal, as cited by Mr. Good). Martial, however, imputes the disease to the chafing of those parts from riding without a saddle. This can never refer to piles. In short, the mariscæ of the poets, the fici and and condylomata of the modern surgeons, are excrescences about the podix, totally different from piles, in cause, appearance, and mode of cure. Wiseman divides them from the piles by a different chapter, in which he gives the various names, and describes some of the tumours as arising at a *distance* from the verge of the anus. Heister and Turner use a similar language. Astruc, as was the fashion in his days, imputes these tumours to syphilis, an error very prevalent till Mr. Hunter's time; but Astruc, who wrote in Latin, is careful to describe them about the *podix*. In short, this distinction was continued with much care from Wiseman to Pott, when the nosologists, none of whom were practical surgeons, undertook the task of *simplifying* science by methodical systems. The business now became, not, as heretofore, to point out certain well marked distinctions between diseases confounded from locality, but requiring a totally different treatment. On the contrary, in order to prove the beauty of simplicity, analogous symptoms were to be noticed, so as to include as many diseases as possible in the same order, class, or genus. These are the misfortunes we derive from complete systems, from nosologies, from dictionaries, from cyclopædias; nor does it appear that we are at all assisted by oriental literature.

An important consideration still remains. The parts subject to these diseases are not only *quæ invitissimus quisque alteri ostendit*, but *quæ invitissimus quisque in altero spectat*. Hence, it sometimes happens, that an unfortunate patient, particularly if a female, supposed to be afflicted with piles, may suffer for months or years an inconvenience which might be removed in as many days. Wherever, therefore, such cases prove obstinate, we advise the young practitioner, instead of referring to a nosological table or an oriental root, to request the patient to examine, by means of a looking-glass, or trust to a confidential friend or servant to make an accurate report.

We are always backward in noticing any grammatical errors or inelegance of expression which do not materially affect the sense. The following, among others that have occurred to us, unnoticed in the errata, we submit to Mr. Good's better judgment in a future edition. *Ulcus*, by some oversight, is made a masculine noun. Under the article Odontia, the reader will recollect O. *senilium*. As we have *puerorum* and *adolescentum*, this must have been intended for *senum*. The same occurs in *salacitas*, where the addition of *α juvenilis* might have led to *β senilis*, instead of *senilium*. The great length of the ERRATA shews the trouble the author had to get his work through the press; and this, when we consider the the complexity of subjects, and the number of languages, cannot surprise us. We are even willing to make a further allowance. We conceive that a work of such labour may have rendered the author weary of perpetual alterations, and have determined him at last to send it to the world with all its faults: and, doubtless, of all these, the gender of a noun, or the awkwardness of a Latin phrase, when neither of them obscure the sense, are the most venial.

*A Treatise on the Physiology and Diseases of the Ear; containing a comparative View of its Structure and Functions, and of its various Diseases, arranged according to the Anatomy of the Organ, or as they affect the external, the intermediate, and the internal Ear.* By John Harrison Curtis, Esq. Aurist to his Royal Highness the Prince Regent, Surgeon to the Royal Dispensary for the Diseases of the Ear, Lecturer on the Anatomy, Physiology, and Pathology of the Ear, Fellow of the Medical Society of London, &c. &c. 8vo. pp. 92. Sherwood and Co.

The modes of book-making are more various than some of our readers are, perhaps, aware of; and it may be thought bad policy in authors to expose one another. But the work now before us is really too bad for us, who have been so long witnesses of this traffic. It would be in vain to express our feelings at seeing the splendid work of our deceased countryman* thus dwindled into a mere vehicle for announcing a new lecturer on the ear. We know not by what name to call this work. A paraphraze, it is true, might be a fair designation, but we are so apt to expect an illustration

---

* For our account of Mr. Saunders's "Anatomy of the Ear, illustrated by a series of Engravings of the natural size, with a Treatise on the Diseases of that Organ, and their proper Treatment," see our xviiith vol. page 5.

in a paraphraze, that we might deceive our readers by such a term; for Mr. Saunders is always perspicuous, and Mr. Curtis, when he ventures to leave his type, is often obscure. We are unwilling to hint at any thing more than obscurity, and have no objection to suppose ourselves mistaken when Mr. C. now and then speaks with great familiarity of a case which the modesty of Mr. Saunders induced him to describe as something remarkable.

It must, however, be admitted that Mr. Saunders is not entirely overlooked by this writer; and we can hardly fail to applaud the manner in which Mr. Curtis contrasts the views of one with the other. "Mr. Saunders's work," he tells us, "is more adapted to the profession than for general reading;" that is, to those who are capable of appreciating its value. Still, however, it would not do for Mr. Curtis to explain things too clearly; he therefore concludes his Preface with the following well-timed invitation.

"The work," says he, "is *necessarily* concise [we presume that he could not venture beyond what he could find in Mr. Saunders]; but any gentleman desirous of farther investigating this interesting subject, may have an opportunity of so doing, by attending the lectures of the author, and the practice of the Royal Dispensary."

But it is time we should explain the manner in which the work is got up.

Mr. Saunders—

"The meatus externus and auricle are sometimes affected with an herpetic ulcerous eruption. It always produces a great thickening of the integuments, and the passage is often so much closed, that a great degree of deafness ensues. The ichor which exudes from the pores of the ulcerated surface inspissates in the meatus, and not only obstructs the entrance of sound, but is accompanied with a great degree of fetor. This disease is not unfrequent."

Mr. Curtis—

"*Herpes.*—Another disease of the external ear, more frequent than the former, is Herpes, or an herpetic eruption, affecting it either in the form of an ulcerous sore, or a thickening of the integuments; and, in the worst cases, these two states are combined, when the passage becomes nearly closed, and temporary deafness is the consequence; the ichorous discharge issuing from the sore, and collected in the passage, hardens and becomes solid, thus obstructing the entrance of sound, and is accompanied also with a disagreeable smell or fetor."

Some remarks follow concerning typhus, and the correction of "constitutional *acrimony*." If we had attempted to keep *our* acrimony in check, the next chapter would, we fear, have undone all.

Mr.

Mr. Saunders—

"The passage of the meatus externus has occasionally been obstructed by an unnatural septum, originating from an elongation or diseased growth of the cutis. I believe these cases are rare, unless the tympanum be diseased, but are not unfrequent after a suppuration and puriform discharge:" of which Mr. S. gives the following instance:—

"J. Hallum applied at the Dispensary for a very considerable and sudden increase of deafness, with which he had been many years afflicted. The deafness had originally been produced by a suppuration of the tympanum, and he recollected that, during the discharge, air had occasionally passed through the meatus in the act of blowing his nose. The discharge had ceased to flow outwardly; and he was no longer capable of forcing air through the meatus. He now spoke of a particular sensation, similar to what people experience when they inflate the tympanum. By placing the patient in the light of the sun, I perceived a septum, which I pierced and lacerated, after which the patient could hear, at nine inches, the tick of a watch, which he was before obliged to place in contact with his ear. Some difficulty arose to prevent the reunion of parts. It was at last accomplished, and the patient's hearing improved to the degree in which it is usually possessed by those who have lost the membrana tympani." P. 27.

Mr. Curtis—

"*Morbid Septum of the Passage.*—An unnatural septum sometimes obstructs the passage of the ear, proceeding from an elongation of the common skin. As the sound is hereby communicated no farther than the external ear, and cannot reach the labyrinth or internal cavity, deafness must be the consequence. But this defect more frequently arises from a diseased tympanum, than from any other cause, where the suppuration is considerable, and much matter has been forced out into the passage.

"The following is the usual progress of the disease:—The patient, after a puriform discharge from the ear, feels a sudden and considerable increase of deafness, to which he has been in a certain degree subject, in consequence of the original complaint. During this original state of deafness, he has been also sensible, on blowing his nose, of air passing at times through the meatus; but, the puriform discharge having now ceased, and the patient being also no longer able, on blowing the nose, to feel air escape through the passage, the existence of a septum becomes undoubted. To this may be added the sensation of a particular fulness of the tympanum.

"If, under these circumstances, the patient be placed in a clear light, and the ear examined, a septum will be perceived. To remove this impediment, the septum is to be pierced and lacerated, when the hearing will be restored to the same degree in which it prevailed under the diseased tympanum, and before the septum was formed.

"So quick is the hearing restored, that, immediately after the operation, operation, the ticking of a watch has been heard at a considerable distance, which could not have been perceived before, even when close to the ear.

"After the operation, much attention is necessary to prevent the closing of the sides of the aperture, and the septum being re-produced."

Mr. Saunders—

On deafness from the accumulation of wax, Mr. S. says, "But the most common impediment to hearing that depends on the state of the meatus externus arises from the inspissation of the cerumen. The quantity which may be collected without impairing the power of hearing, cannot easily be determined. In many persons the quantity is naturally considerable; but, unless its proper consistence be altered, the functions of the passage are not much injured, whereas a small portion of inspissated cerumen lodged in the membrana tympani, will deprive a person of his hearing. The symptoms which are attached to an inspissation of the cerumen are pretty well known. The patient, besides his inability to hear, complains of noises, particularly a clash or confused sound in mas ication, and of heavy sounds like the ponderous strokes of a hammer, &c." P. 23.

Mr. Curtis—

"*Inspissated Cerumen.*—The most frequent cause of deafness, connected with the state of the external passage, is that arising from collected cerumen or wax; a due secretion of the passage is absolutely necessary to keep it in a healthy condition, as well as to preserve it from external injury. A defective, or too profuse, secretion is equally the cause of deafness, and the cerumen frequently becomes indurated and inspissated to such a degree as to cause obstinate dullness of hearing. The actual quantity to produce this effect cannot be determined: in many persons the secretion is very copious, compared with what it is in others; but, unless it be considerably altered by stagnation, it does not seem materially to affect the functions of the part. When accumulated on the membrane of the tympanum, it frequently obstructs the sense of hearing.

"The symptoms that particularly mark this complaint are the following:—With the general sense of deafness, there is combined the impression of noises in the ear, consisting either of a particular clash or confused sound, or a heavy sensation like the noise of a hammer: these sounds prevail most while eating."

And now for our friend Juvenis, who, we can assure him, is never entirely out of our mind. Let us advise him to go to the fountain-head, and consult Saunders and other original writers, when he wishes for information. But, most of all, let us advise him to remember the poor author mentioned by our English Aristarchus, and the fate he underwent from clipping and coining. There are already books enough in the world, without such a mode of multiplication. But do not let him suppose that, by purchasing Mr. Curtis, he may save the expence of Mr. Saunders. The plates in the latter

 are are invaluable, and, without them, the anatomical part is useless. Mr. Curtis, it is true, has a frontispiece of "Acoustic Instruments,"—some which, we conceive, would be very likely to obstruct the passage of sound into the natural ear.

---

*Nouveaux Elemens de Therapeutique et de Matiere Medicale, &c. &c.*—*New Elements of Therapeutics and Materia Medica, &c.;* bv S. L. ALIBERT, Knight of several Orders, Consulting Physician to the King, &c. &c. 2 vols. 8vo.

IT will hardly be believed that the French are still without a regular Pharmacopœia and Materia Medica. It is now nearly eighty years since the codex was framed, since which period medicine has risen from an art to a science, and chemistry only resembles what it was eighty years ago in the name. Much praise is, therefore, due to the Colleges of London and Edinburgh, in revising, from time to time, the formulæ according to the progress of the physical sciences; for want of this in France, legal prescriptions are unknown, and medical men can invent and change them at their pleasure. Authors, who had required, or fancied they merited, reputation, have, from time to time, given treatises on the Materia Medica and *(l'Art de Formula)* the Art of compounding Medicines. In the greater part of them, the profoundest ignorance is observable. Every man had a few nostrums of his own, invented he knew not why, and which operated he scarcely knew how. In this state was therapeutics in France, when M. Alibert, a man of genius and application, undertook to remedy the defect. To succeed entirely, perhaps, he would have done well to have consulted the British Pharmacopœias; but the study of medicine in England and in France is entirely different; and duly to appreciate the superiority of a foreign medicine over the prescriptions in ordinary use, the physician must have resided in that country and practised physic with success for years on the principles there adopted. M. Alibert commences with prolegomena as an introduction to the study of therapeutics and materia medica. In these he announces his design.

"When the celebrated Stahl changed the face of practical medicine, he offered up the most ardent prayers that therapeutics might be freed from those dark and false theories which diverted the art of healing from its sublime destinies. 'I should like, (said he,) with a bold hand, to cleanse this Augean stable.'" He confesses that he only submitted in murmuring to the yoke of Sylvius.

4 M. Alibert

M. Alibert adds, "I have now dared to essay the task indicated by this great man,—I have dared to penetrate this science, peopled with errors, in which the language is as defective as the ideas, where all is to be reorganized, the principles and the matter. It is true, that a happy concurrence of circumstances has favoured my zeal and sustained my efforts. I write at a period of the science, when anatomy, physiology, chemistry, mineralogy, and botany, have been illustrated by immense progress, and in which philosophical methods have prepared this branch of our art for the numerous reforms it ought to undergo."

Such is the object of M. Alibert, and he has adopted the best method of accomplishing it, by following a regular system of analysis of all the medical agents: he first gives the French or Latin names, next the natural history of the substance, and then follow, in regular order, its physical properties, its chemical properties, its medical properties, and, lastly, the mode of administering it. This method is attended with several advantages: the student sees at one view all he can desire to know as to the nature of the ingredients of which he composes his medicines.

But the work has another important property; it contains a French Pharmacopœia, which the English student would do well to study. The French system of medicine is, in some respects, far more simple than ours, that is, it makes greater use of diets, ptisans, simple decoctions, and infusions of indigenous herbs or flowers; and, if the French physician relies too much on them, probably the English are too inattentive in their instructions to nurses. We, therefore, strongly recommend this work to the English student as a part of his library of reference.

---

*De la Goutte et des Maladies Goutteuses.—On the Gout and Gouty Disorders, &c.* Par M. Guilbert, M.D. &c. &c. &c. 1 vol. 8vo. 1817.

The volume before us contains a copious history of the Gout; the authors who have treated the subject; and the remedies that have been, from time to time, recommended; with an analysis of the latter, and judicious remarks on their merits.

The gout, like many other disorders, requires, if not a new appellation, at least an abandonment of the old one. M. Guilbert informs us, that the gout has apparently been so called, because it was supposed to be a catarrhal disorder; and that the gouty matter fell *goutte à goutte* (drop by

 drop)

drop) on the place affected. The English have adopted the term without any reference to the meaning, and, consequently, had it expressed a just idea, that idea would have been lost to us, as has really happened in words imported into our language; we need only adduce the plant leontodon, or *dens leonis:* the French have named it by translating the term *dent de lion;* whereas, in our language, dandelion is merely a word without either sense or meaning.—Why, in the name of science, we will ask, is not the name consecrated by Hippocrates restored (*Arthritis,* pain in the joints)? it may be objected that it only describes the effect and not the cause of the disorder; but fever, and, in fact, two-thirds of the medical nomenclature, are under the same predicament; and, until we have a scientific name exactly appropriate, let us for ever banish the ridiculous unmeaning term, or, if it do mean any thing, the false term Gout for that of Arthritis. To return, M. Guilbert has bestowed great pains on the compilation of this volume, which is a separate impression of the article in the Dictionnaire des Sciences Medicales, of which he is one of the compilers. He has given a fair and candid review of the various theories and prescriptions for the gout, which was probably better than if he had attempted to explore the nature of a disorder which he conceives has hitherto defied the skill of all physicians, ancient and modern. Palliatives may be administered, and occasional cures obtained, and rules prescribed as to regimen, which are better than medicine; but certain it is, that to this hour no general remedy has been discovered. Considerable light has, indeed, been recently thrown on the subject by the English, and, it is greatly to be regretted, that M. Guilbert did not avail himself of the free intercourse with England to procure the best and latest works on the subject, which necessarily leaves his work imperfect. We were much disappointed in finding no notice taken of Dr. Kinglake, Mr. Want, Dr. Scudamore, and some others, who have given their opinions on the late improvements in the treatment of gout: but most of all, that our illustrious Sydenham should be so ill understood, since whose time nothing has been added to the history of the disease. Our improved pathology has, indeed, instructed in the proximate cause of the pain and other symptoms, whilst the boldness of some late remedies has given us courage in the treatment of a complaint with which our immediate predecessors were fearful of interfering.

